# Naproxen-Loaded Poly(2-hydroxyalkyl methacrylates): Preparation and Drug Release Dynamics

**DOI:** 10.3390/polym14030450

**Published:** 2022-01-23

**Authors:** Abeer Aljubailah, Saad M. S. Alqahtani, Tahani Saad Al-Garni, Waseem Sharaf Saeed, Abdelhabib Semlali, Taieb Aouak

**Affiliations:** 1 Chemistry Department, College of Science, King Saud University, Riyadh 11451, Saudi Arabia; akaljubailah@imamu.edu.sa (A.A.); tahanis@ksu.edu.sa (T.S.A.-G.); wsaeed@ksu.edu.sa (W.S.S.); 2 Groupe de Recherche en Écologie Buccale, Faculté de Médecin Dentaire, Université Laval, Quebec City, QC G1V 0A6, Canada; abdelhabib.semlali@greb.ulaval.ca

**Keywords:** poly(hydroxyalkylmethacrylate)/Naproxen, drug release, solubility enhancement, drug-polymer miscibility, cell adhesion, toxicity, performance comparison

## Abstract

Poly(2-hydroxyethylmethacrylate)/Naproxen (NPX/pHEMA) and poly (2-hydroxypropyl methacrylate)/Naproxen (NPX/pHPMA) composites with different NPX content were prepared in situ by free radical photopolymerization route. The resulted hybrid materials were characterized by Fourier transform infrared spectroscopy (FTIR), differential scanning calorimetry (DSC), scanning Electron microscopy (SEM), and X-ray diffraction (XRD). These composites have been studied as drug carrier systems, in which a comparison of the in vitro release dynamic of NPX between the two drug carrier systems has been conducted. Different factors affecting the performance of the release dynamic of this drug, such as the amount of Naproxen incorporated in the drug carrier system, the pH of the medium and the degree of swelling, have been investigated. The results of the swelling study of pHEMA and pHPMA in different media pHs revealed that the diffusion of water molecules through both polymer samples obeys the Fickian model. The “in vitro” study of the release dynamic of Naproxen from NPX/pHEMA and NPX/pHPMA drug carrier systems revealed that the higher percentage of NPX released was obtained from each polymer carrier in neutral pH medium, and the diffusion of NPX trough these polymer matrices also obeys the Fickian model. It was also found that the less the mass percent of NPX in the composites, the better its release will be. The comparison between the two drug carrier systems revealed that the pHEMA leads to the best performance in the release dynamic of NPX. Regarding Naproxen solubility in water, the results deducted from the “in vitro” study of NPX/pHEMA10 and NPX/pHPMA10 drug carrier systems revealed a very significant improvement in the solubility of NPX in media pH1 (2.33 times, 1.43 times) and 7 (3.32 times, 2.60 times), respectively, compared to those obtained by direct dissolution of Naproxen powder.

## 1. Introduction

Medications are introduced into the human body through various drug delivery routes. For example, administered orally (by mouth), intravenously, intramuscularly, or breathed into the lungs (inhaled). The oral route remains the most popular way of drug administration [1]. In fact, it is the most preferred route by patients due to the ease of self-administration, pain avoidance, and cost-effectiveness. Some other advantages are that the oral ingestion route provides, for the patients, the least amount of sterility constraints, the minimal possibility to introduce systemic infection as a complication of treatment, the versatility to accommodate various types of drugs, and, most importantly, high patient compliance. Hence, it is the most employed route of drug delivery [2,3]. However, an orally administered drug must reach its target site at a concentration sufficient to induce the desired therapeutic effect. The term “bioavailability” refers to the fractional extent to which an administered dose of drug reaches its site of action or bloodstream from which the drug has access to finally reach its site of action [4,5]. Although the definition applies to any route of administration, practically-speaking the term is usually used for the oral route [6]. A severe drawback of oral ingestion of drugs is the limited absorption of some drugs due to their physical characteristics (e.g., poor aqueous solubility and low membrane permeability) [4]. According to the biopharmaceutical industry, all drugs must meet certain minimal requirements to achieve clinical effectiveness. More than 40% of newly discovered chemical entities entering the drug development pipeline fail to reach therapeutic range due to their poor water solubility, which in turn influences the absorption of the drug from the gastrointestinal tract, thereby leading to low bioavailability [7]. Among the severe drawbacks of oral drug administration is that, in some cases, a significant portion of the drug is destroyed in the stomach (very acidic pH) before reaching the intestines (neutral pH) where it will be absorbed, and, therefore, additional amounts of drug are required to reach the therapeutic threshold, not to mention the side effects that can cause fragments resulting from the degradation of these drugs. Other medications cause direct irritation to the gastric mucosa due to the inhibition of prostaglandins and prostacyclins and thus causes ulceration, epigastric distress, and/or hemorrhage [8]. In order to minimize these inconveniences, several authors have run towards the encapsulation of these drugs in the form of intelligent systems labeled as “drug-carrier” acting according to the environment where they are found. Sustained release of aspirin formulation would reduce the undesired side effects, reduce frequency of administration, and improve patient compliance [9]. Zheng et al. [10] investigated the ibuprofen/montmorillonite intercalation composites as the drug release system, and the in vitro results revealed that the release of ibuprofen from this system was affected by the pH value of the dispersion. The release rate in simulated intestinal fluid (pH = 7.4) was noticeably higher than that in simulated gastric fluid (pH = 1.2).

Polymers have played a key role in the advancement of drug delivery technology. The “in vitro” release of ibuprofen was also investigated by Carreras et al. [11]. These authors encapsulated this drug by poly(ε-caprolactone) using the solvent casting method. The results obtained revealed that the system has low homogeneity in particle size distribution, with a particle size average of 846.9 nm, and, therefore, they are microspheres. Mangindaan et al. [12] developed a controlled release system composed of surface modified porous polycaprolactone (PCL) membranes combined with a layer of tetraorthosilicate (TEOS)–chitosan sol–gel. The drugs chosen in this investigation were silver-sulfadiazine (AgSD) and ketoprofen, which were impregnated in the TEOS–chitosan sol–gel, and the results obtained revealed that the release of AgSD on O_2_ plasma-treated porous PCL membranes was prolonged when compared with the pristine sample. On the contrary, the release rate of ketoprofen revealed no significant difference on pristine and plasma-treated PCL membranes. Diclofenac sodium(DS) combined with an electrospinning nano-and-nanofiber (DS-NNEM) mesh of polycaprolactone (PCL)-chitosan (CH) prepared by the electrospinning technique was the subject of a controlled release study of soluble DS in water [13]. Because of the very slow degradation of nanofiber mats, these authors suggested that DS is released either by diffusion or by permeation through DS-NNEMs structure. In summary, the results suggest that NNEMs technology can potentially serve as a biomimetic platform for loading and the sustained release of biologically active therapeutic compounds and other drugs for prolonged periods.

**Scheme 1:**
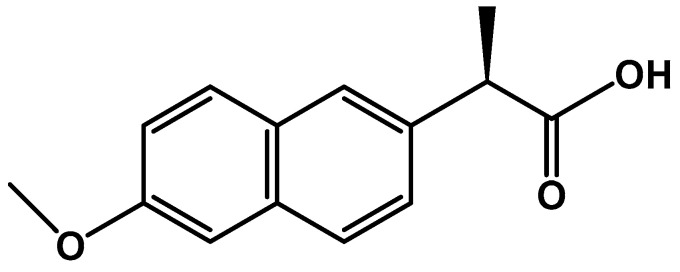
Chemical structure of Naproxen.

Naproxen (NPX), also known by its trade name “Proxen” (Scheme 1), belongs to the family of aryl propanoic acids such as ibuprofen, ketoprofen and diclofenac. This medication is a potent nonsteroidal anti-inflammatory drug (NSAIDs) that is used to treat acute pain, inflammation, as well as pain related to arthritis and rheumatic diseases [14,15]. However, the pharmaceutical applications of Naproxen is hampered by its poor water solubility [16]. Naproxen is a weak acid drug (pKa 4.2) that belongs to BCS class II drugs. It is a highly lipophilic drug (log P 3.18) with an aqueous solubility of 0.0159 mg·mL^−1^ at 25 °C [15,17].

Among the polymers that have attracted the attention of many researchers in the biomedical field, poly(2-hydroxyethyl methacrylate)(pHEMA) was found as a suitable compatible biomaterial [18,19] and a good candidate for drug delivery [20,21,22,23,24,25,26] and bone implantation [27,28,29,30]. This material can be prepared by bulk polymerization with low water content or by suspension polymerization to form microbeads [31,32]. pHEMA is usually reported to be biocompatible but less biodegradable [33]. However, for oral administration of this polymer, it must not be biodegradable.

Although pHEMA has been extensively studied in the biomedical field, its analog with additional methyl group, poly (2-hydroxypropyl methacrylate)(pHPMA) is very little known in this field. Therefore, it will be curious to know the reasons why this polymer has not been able to place itself among polymers selected as potential candidates as a carrier in drug delivery or as scaffolds used in the biomedical field.

In order to have an idea on the performance of pHPMA in the drug release domain, a comparative study on the effect of the 2-hydroxyalkyl methacrylate substituent on the release dynamics of Naproxen from the Naproxen/poly (2-hydroxyalkyl methacrylate) drug carrier system was carried out. To reach this goal, two series of composites involving Naproxen combined with pHEMA and Naproxen combined with pHPMA as polymer composites were prepared with different compositions by solvent casting route. The distribution of NPX particles in the resulted systems were studied by FTIR, DSC, XRD, and SEM methods, while the cell viability and the cell adhesions were examined by MTT test and LDH essay. A comparative study of the efficiency of these two drug carrier systems was carried out on the release dynamic of Naproxen by varying different parameters that affect the release performance of NPX, such as the percentage of medication incorporated in the polymer matrix and the pH media. The improvement in the solubility of Naproxen in the different pH media was also deduced from the release process.

## 2. Materials and Methods

### 2.1. Chemicals

HEMA (purity, ≥99%), HPMA (purity, ≥99%), and AIBN (purity, 98%) were provided by Sigma Aldrich (Taufkirchen, Germany). Proxen tablets manufactured by GRUNENTHAL were purchased from Riyadh Pharma (Saudi Arabia). Monomers were purified from hydroquinone (inhibitor) by distillation under reduced pressure. AIBN was purified three times by dissolution and recrystallization in ethanol. Human oral cancer cell line Ca9-22 cells were obtained from the laboratory of Dr. Abdelhabib Semlali (GREB–laval University, Quebec City, QC, Canada). RPMI-1640 medium was purchased from ThermoFisher (Burlington, ON, Canada), and the fetal bovine serum (FBS, Gibco) and 1% penicillin/streptomycin solution were from Sigma Aldrich (St. Louis, MO, USA).

### 2.2. Naproxen Extraction

Commercialized Proxen 500 mg tablets were ground into a fine powder using an electric grinder. The Proxen powder obtained is added to a 3M HCl solution and stirred for 24 h then left to stand for 1 h. Naproxen (NPX) or (S)-2-(6-methoxynaphthalen-2-yl) propionic acid, which is very poorly soluble in water precipitates, the additives dissolve, and the precipitant is then recovered by filtration. The extracted NPX powder is washed several times with water and then dried under a vacuum at 25 °C to constant weight. In order to remove the residual matter from the organic phase, the dry precipitate obtained is dissolved in chloroform and then transferred to a separating funnel containing an equivalent amount of distilled water. The whole is then stirred until the complete dissolution of Naproxen. The two phases are finally separated by settling. This process was repeated three times to ensure the purification of the product. Pure Naproxen is then extracted from the isolated organic phase by evaporating chloroform at room temperature (25 °C) using a rotary. The melting point of the pure NPX white crystals obtained, measured by DSC analysis, indicates 166 °C, which agree with the literature [34].

### 2.3. Preparation of NPX/pHEMA and NPX/pHPMA

NPX/pHEMA and NPX/PHPMA composites containing 2, 5, 7, and 10 wt% of NPX were prepared in situ by free radical polymerization at 25 °C in the presence of NPX using camphorquinone as a photoinitiator. Using known amounts distillated under reduced pressure of HEMA and HPMA monomers, camphorquinone and NPX were weighed with precision and placed in a Teflon pan traversed with a stream of nitrogen U. These mixtures are irradiated throughout the reaction time by means of UV light coming from a UV lamp with a wavelength of 380 nm and a power of 13.3 MW. A solid film deposed in the Teflon pan is obtained indicating the completion of the polymerization reaction. To remove all traces of residual monomer encrusted in the film obtained, the Teflon pan and polymer film set are placed in a vacuum oven maintained at 40 °C until constant mass. The aggregated NPX particles deposited or glued to the film surface are removed by washing three times with distillated water. Two series of NPX/pHEMA and NPX/pHPMA mixtures containing 2, 5, 7, and 10 wt% of NPX content are prepared by this same method, and the preparation conditions are summarized in Table 1.

### 2.4. Characterization

The FTIR spectra of NPX powder, PHEMA homopolymer, NPX/PHEMA, and NPX/PHPMA composites films were performed in the wavenumber range 400–4000 cm^−1^ on a Nicolet 6700 FT-IR from the company Thermo Scientific. A 30,000–200 cm^−1^ diameter diamond-like Smart orbit crystal reflector, supplied by the same company, was used to accomplish this task. The DSC thermograms of drug, polymer, and their mixtures were performed on a Shimadzu DSC-60 (Japan) previously calibrated with indium. An amount of 8–10 mg of NPX powder or film samples were deposited in an aluminum pan and then closed, being placed in the DSC analysis cell. All samples were scanned from −40 to +240 °C under nitrogen gas atmosphere at a heating rate of 20 °C·min^−1^. All the thermograms taken from the second scan run revealed no traces of polymers or drug degradation. The *T_g_* value of pure constituents or their mixture was taken precisely as the median point on the thermogram indicating the variation in the heat capacity versus the temperature. The *T_m_* value was taken exactly at the top of the endothermic peak. The surface morphology of NPX particles, polymers, and their composites were examined by scanning electronic microscope using a JEOL JSM-6360LV SEM (Tokyo, Japan) at an accelerating voltage of 10 kV. The surface and cross-sections of samples were sputter-coated with a thin layer of gold prior and was imaged at a magnification range of 300–3000 nm. The crystalline structures of NPX powder, polymers, and mixtures were examined by XRD analysis on an X-ray diffractometer (Rigaku D/max 2000) equipped with a Cu anode tube. The applied voltage was 40 kV and a generator current of 100 mA. All samples were examined at 2*θ* = 5°–80° at a scanning rate of 1.0°·min^−1^.

A U-2910 spectrophotometer manufactured by Hitachi Company was used to measure ultraviolet and visible light absorbance of NPX released. Absorbance was measured using quartz cuvettes with a side length of 1 cm. The wavelength corresponding to the maximum absorbance of NPX was 230 nm. The released NPX concentration was deduced from a linear calibration curve indicating the change in absorbance versus concentration.

### 2.5. Cell Culture and Proliferation Assessment

The human oral cancer cell line Ca9–22 cells were cultured at 37 °C and 5% CO_2_ in RPMI-1640 medium (Thermo Fisher Scientific, Burlington, ON, Canada), supplemented with L-glutamine, 5% fetal bovine serum (FBS, Gibco) provided by the same company, and 1% penicillin/streptomycin solution (Sigma-Aldrich, Oakville, ON, Canada). Cell proliferation was bi-evaluated using two MTT and LDH tests as described by Semlali et al. 2021 [35,36] and Contant et al. 2021 [37]. For the MTT assay, 10^5^ Ca9-22 cells per well containing the sample were seeded in 24-well plates for 24 h. After adhesion and growth of the cells, the culture medium is replaced by a new one containing a solution of MTT at 5 mg∙ml^−1^ in PBS and left for 3 h at 37 °C in the dark. Then, the cells were lysed with HCl, 0.05 N, in 1 mL of isopropanol. Addition of 100 µL of analysis buffer to test wells from 96-well microplates was required to measure absorbance at 550 nm by an iMark reader (Bio-Rad). The percentage of viable proliferating cells was determined using Equation (1)

(1) $$Cell.viability(\%)=\frac{OD_{T}-OD_{B}}{OD_{C}-OD_{B}}\times 100,$$

where *OD_T_*, *OD_B_,* and *OD_C_* are the optic densities of the treated cell, blank, and control, cell respectively.

Adhesion positive control was the plate for the treated cultured tissues. The negative control for cell adhesion was the plate for untreated cultured tissue. LDH assay was realized by the LDH Cytotoxicity Detection Kit from Roche, which allows to directly quantify the cell death in culture based on the measurement of lactate dehydrogenase released into growth media. As described in our previous work [35,36], 10^5^ cells per well were seeded in 24-well plates containing NPX/pHEMA and NPX/pHPMA with different compositions. After adhesion for 24 h, 50 μL of each supernatant was transferred in triplicate into a 96-well plate and supplemented with 50 μL reconstituted substrate mixture. Then, the plates were incubated for 30 min at room temperature in the dark until the yellow color developed, before reading at 490 nm with an xMark microplate absorbance spectrophotometer (Bio-Rad, Mississauga, ON, Canada). Triton X-100 (1%) was used as a positive control for LDH, and the negative one was obtained with untreated cells. LDH release activity was calculated using Equation (2)

(2) $$LDH.activity(\%)=\frac{ABS_{p}-ABS_{nc}}{ABS_{pc}-ABS_{nc}}\times 100,$$

where *ABS_p_*, *ABS_nc_*, and *ABS_pc_* are the absorbance of drug-carrier system, positive control, and negative control, respectively.

### 2.6. Swelling Properties

The swelling behavior of pHEMA and pHPMA hydrogels was studied on samples of thin films of dimensions 3 cm × 3 cm and thickness varying between 2.20 and 2.56 mm. Each film sample of determined mass (*m_o_*) was placed in 50 mL of an aqueous solution at known pH (1 or 7) and maintained at 37 °C, then stirred (260 rpm) until the swelling equilibrium was reached. The mass of the medium absorbed at each time interval (*m_t_*) is obtained by weighing the film after delicately wiping the droplets deposited on the two surfaces using tissue paper. The swelling degree of pHEMA and pHPMA film samples was determined from Equation (3).

(3) $$S(wt\%)=\frac{m_{t}-m_{o}}{m_{o}}\times 100,$$

### 2.7. Density Measurements

Polymer density values were determined at 25 °C using a pycnometer, in which cyclohexane was used as a non-solvent and Equation (4) [38]:

(4) $$\rho =\frac{m_{p}\times \rho_{chx}}{m_{p}+m_{pc}+m_{T}},$$

where *m_p_* is the mass of the polymer, *m_pc_* is the mass of the pycnometer with cyclohexane, and *m_T_* is the mass of the pycnometer with cyclohexane and polymer. $\rho_{chx}$ is the density of cyclohexane (0.78 g∙cm^−3^). Each experiment was triplicated, and the density was taken from the average arithmetic values obtained.

### 2.8. In Vitro Release Dynamic of NPX

The “in vitro” release dynamic of NPX from the NPX/pHEMA and NPX/pHPMA drug carrier systems was investigated at body temperature (37 °C) in aqueous media of pH1, 3, 5, and 7. NPX released was monitored for 72 h, in which 0.5 mL of the solution was withdrawn after each time interval, then dosed by UV analysis. The accumulative drug release percent ADR (wt %) at time *t* was calculated at chosen time intervals using the following equation:

(5) $$ADR(wt\%)=\frac{m_{t}\times 100}{m_{o}},$$

where $m_{t}$ and *m_o_* are the total mass of NPX released at a certain time *t* and the initial mass of drug loaded in the polymer.

## 3. Results and Discussions

### 3.1. Characterization

#### 3.1.1. FTIR Analysis

**Figure 1:**
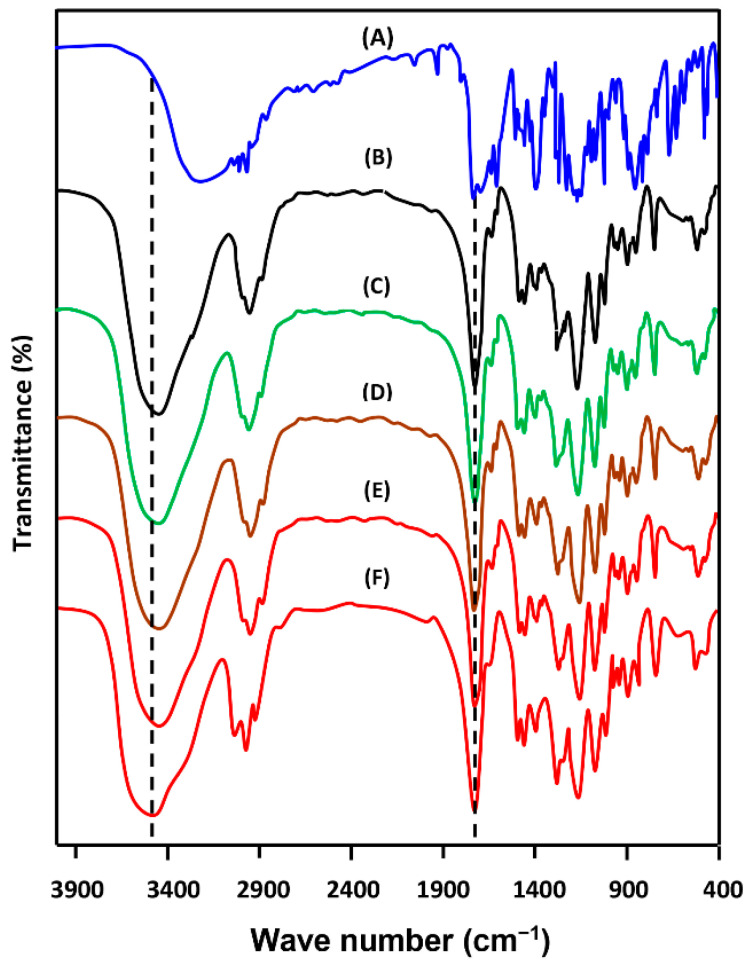
Comparative FTIR spectra of: (**A**) pure NPX; (**B**) NPX/pHEMA2; (**C**) NPX/pHEMA5; (**D**) NPX/pHEMA7; (**E**) NPX/pHEMA10; (**F**) virgin pHEMA.

**Figure 2:**
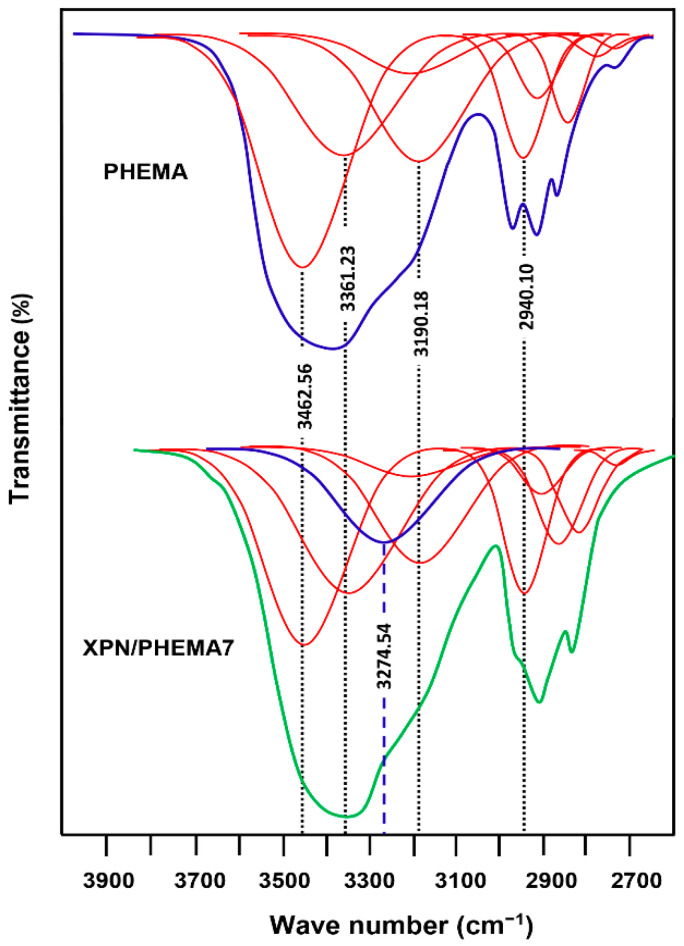
Deconvolution in Lorentzian of the FTIR spectra of pure pHEMA and NPX/pHEMA7 composite spectra between 4000 cm^−1^ and 2600 cm^−1^.

**Figure 3:**
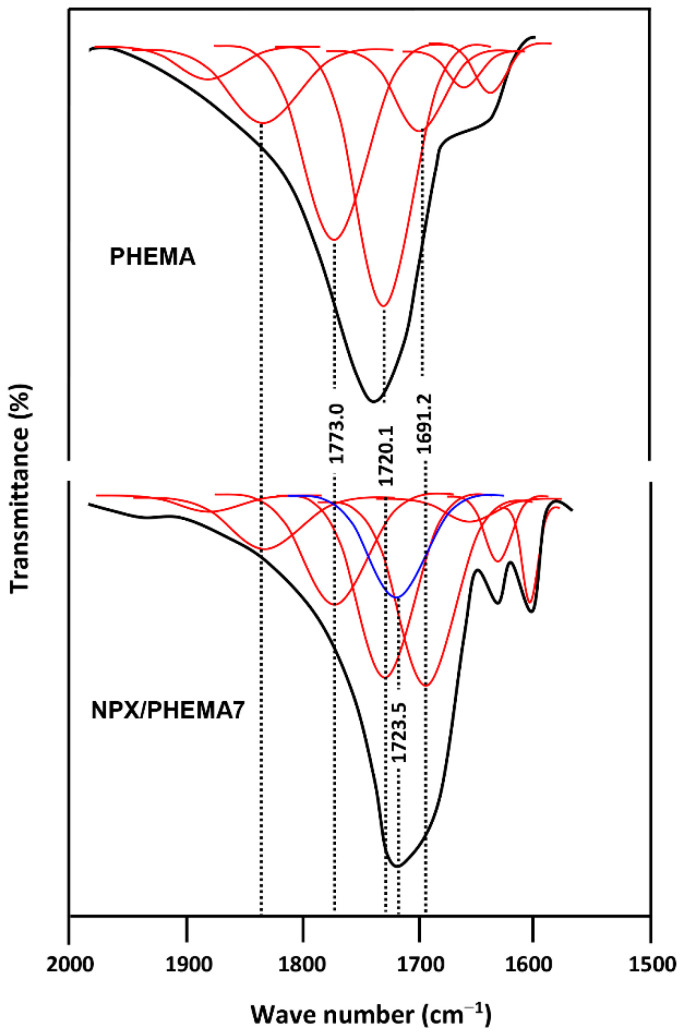
Deconvolution in Lorentzian curves of the FTIR spectra of pure pHEMA and NPX/pHEMA7 composite spectra between 2000 cm^−1^ and 1600 cm^−1^.

**Figure 4:**
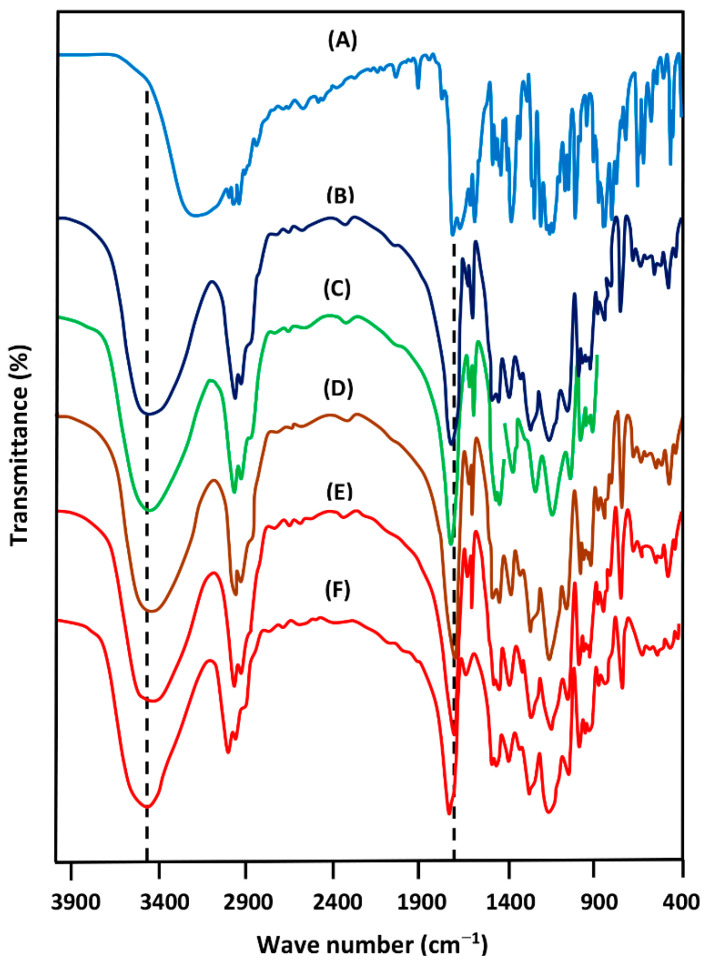
Comparative FTIR spectra of: (**A**) pure NPX; (**B**) NPX/pHPMA2; (**C**) NPX/pHPMA5; (**D**) NPX/pHPMA7; (**E**) NPX/pHPMA10; (**F**) virgin pHPMA.

A comparison between the FTIR spectra of the NPX/pHEMA composites with those of their components shown in Figure 1 reveals, for the composite absorption bands, that they are practically similar to those of pure pHEMA. The NPX/pHEMA spectra exhibits a shift in the broad absorption band of the hydroxyl group vibrations from 3472 to 3463 cm^−1^ and a shift of the sharp absorption band attributed to the carbonyl group (C=O) vibration of pHEMA from 1727.62 cm^−1^ to 1724.32 cm^−1^. It is well known that the position of the vibration peak of the carbonyl group suggests that most of the carboxylic acid groups are associated with the intermolecular hydrogen bonds formed between the HEMA derived moieties and the acid groups [39,40,41]. In addition, the wide absorption band in the spectral region 3200–3600 cm^−1^ corresponding to the vibrations of the OH group also confirms that hydrogen bonds form in the structure of poly(2-hydroxyethyl methacrylate-co-acrylic acid). The deconvolution in Lorentzian peaks of the hydroxyl absorption band between 2600 cm^−1^ and 4000 cm^−1^ (Figure 2) also reveals the appearance of a new band at 3274.54 cm^−1^, attributed to the vibration of the hydrogen bond between hydroxyl group of pHEMA and carbonyl group of NPX. On the carbonyls side, the deconvolution of the absorption band between 1600 cm^−1^ and 2000 cm^−1^ (Figure 3) reveals another new absorption band at 1723.5 cm^−1^, thus confirming this finding. Similar results are also observed for NPX/pHPMA composites in Figure 4. Indeed, the comparison between the FTIR spectra of the NPX/pHPMA composites with that of its pure polymer reveals a small shift in the absorption band of the carbonyl group of pHPMA toward the lower wave number (from 1724.12 cm^−1^to 1727.34 cm^−1^) and in the hydroxyl group (from 3400.07 cm^−1^ to 3395.36 cm^−1^). These facts are without doubt attributed to a dynamic caused by the hydrogen bond interactions leading to a miscibility of these two components.

#### 3.1.2. XRD Analysis

**Figure 5:**
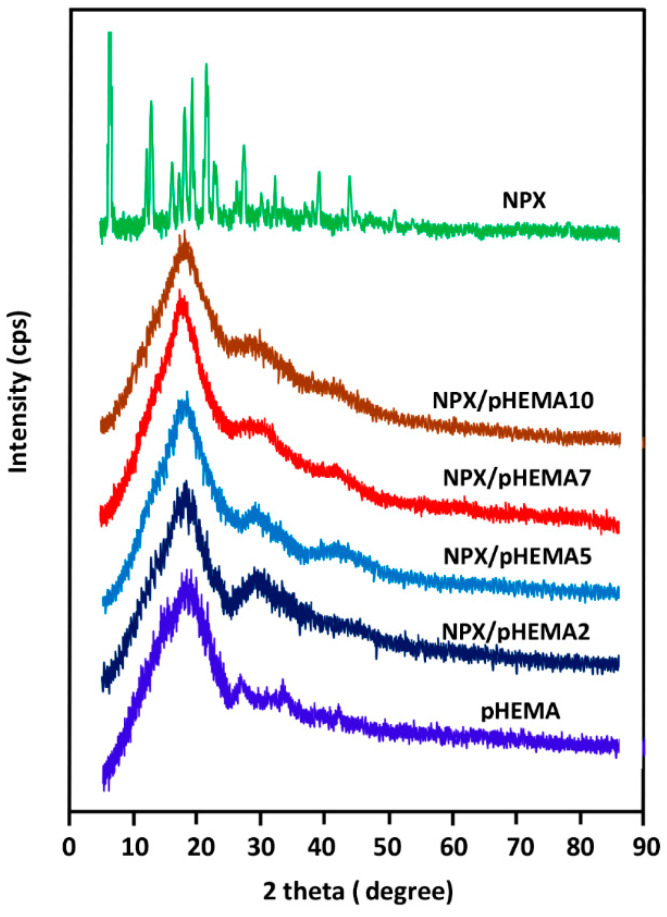
X-ray diffraction spectra of NPX, pHEMA, and NPX/pHEMA systems with different NPX contents.

**Figure 6:**
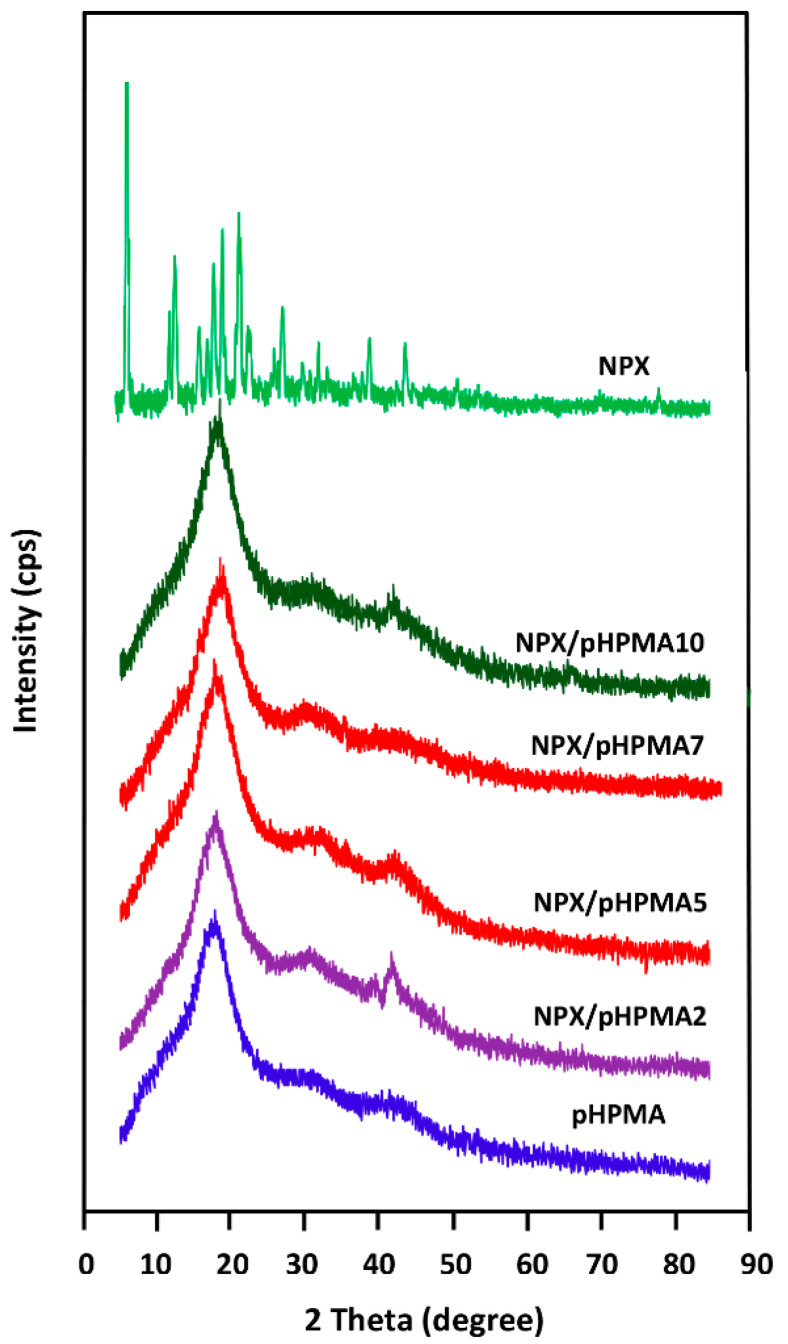
X-ray diffraction spectra of NPX, pHPMA, and NPX/pHPMA systems with different NPX contents.

The crystalline structure of NPX in the pHEMA and pHPMA polymer matrices was investigated by X-ray diffraction, and the results obtained are gathered with their pure components in Figure 5 and Figure 6, respectively. As shown in Figure 5, the XRD pattern of pure NPX aggregated powder shows its highly crystalline structure and nature through the distinct peaks at 6.5°, 12.4°, 16.6°, 19°, 20°, 22.5°, 24°, and 28.6° 2*θ*, which are in good agreement with the literature [42,43]. The XRD spectra of pHEMA and pHPMA reveal an amorphous structure. The XRD motif of the NPX/pHEMA7 composite shows no new crystallinity signals or those characterizing the crystallinity of pure NPX; as for the pure polymer, this material exhibits a completely amorphous structure. Similar results are also obtained for the NPX/pHPMA systems as shown in Figure 6. This indicates that the NPX drug is uniformly distributed in its molecular level inside the polymer matrix.

#### 3.1.3. DSC Analysis

**Figure 7:**
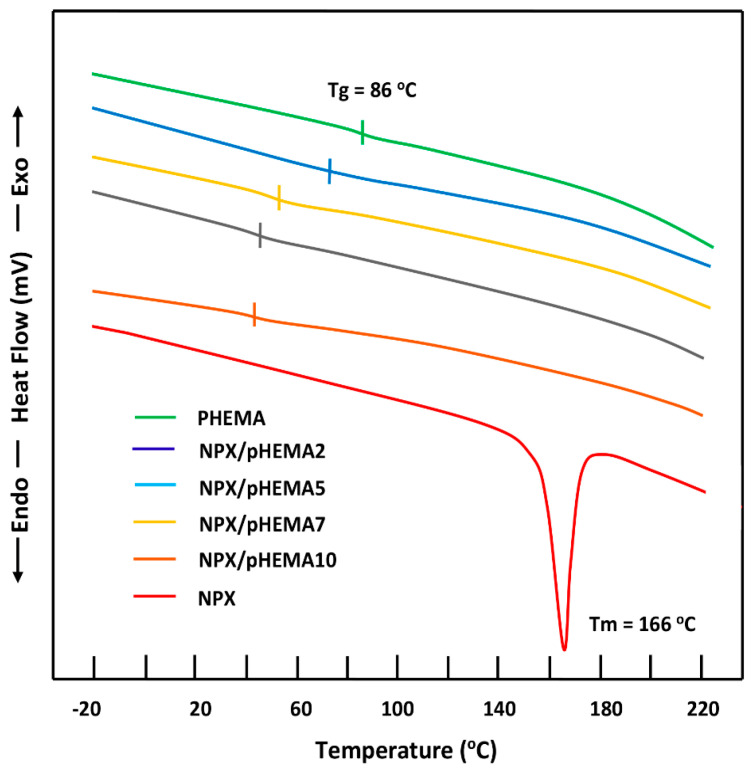
DSC thermograms of pure NPX, pHEMA, and NPX/pHEMA7 systems.

The DSC thermograms of pure NPX, pHEMA, and NPX/pHEMA systems with different NPX contents are shown in Figure 7. The thermal curve of pure pHEMA shows a glass transition temperature (*Tg*) at 86 °C, which agrees with that of the literature [44], while the pure Naproxen shows, through its thermal plot, a sharp endothermic peak at 166 °C characterizing its melting temperature [34]. The NPX/pHEMA thermograms reveal a small shift in the *Tg* of pHEMA toward the low temperatures and a complete disappearance of the NPX melting peak. This reveals a uniform distribution of the NPX filler in the polymer matrix in its molecular level, in which Naproxen loses its crystallinity, thus confirming the results obtained by FTIR and XRD analysis. The decrease in the *Tg* value of the polymer in the mixture is probably due to the increase of the free volume between the polymer chains caused by the insertion of MPX molecules between them, thus promoting the chain sliding.

**Figure 8:**
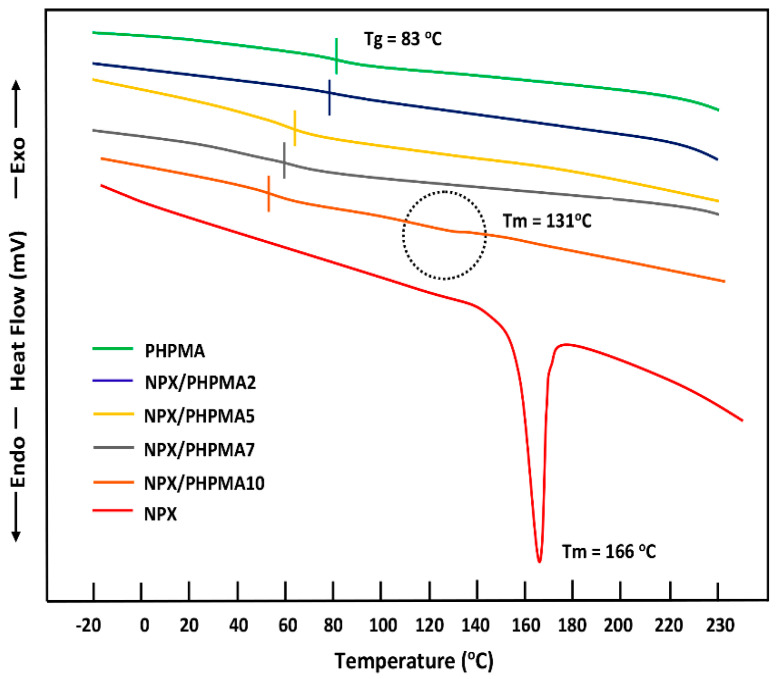
DSC thermograms of NPX, pHPMA, and NPX/pHPMA systems with different NPX contents.

The thermal analysis of NPX/pHPMA systems by DSC technique led to the results of Figure 8. As it can be observed on the thermogram of pure pHPMA, a transition appears at 83 °C characterizing the glass transition of this polymer [45]. Concerning the NPX/pHEMA system, as for the system containing PHEMA carrier, a shift was shown in the Tg of the pHPMA from 82 °C to 54 °C as the NPX content in the mixture increased. A complete disappearance of the transition characterizing the fusion of the NPX is also observed on the thermograms of NPX/pHPMA mixtures, except that containing 10 wt% of NPX content, in which a weak transition at 131 °C attributed to the melting point of excess of NPX aggregates.

### 3.2. Cells Adhesion and Toxicity

**Figure 9:**
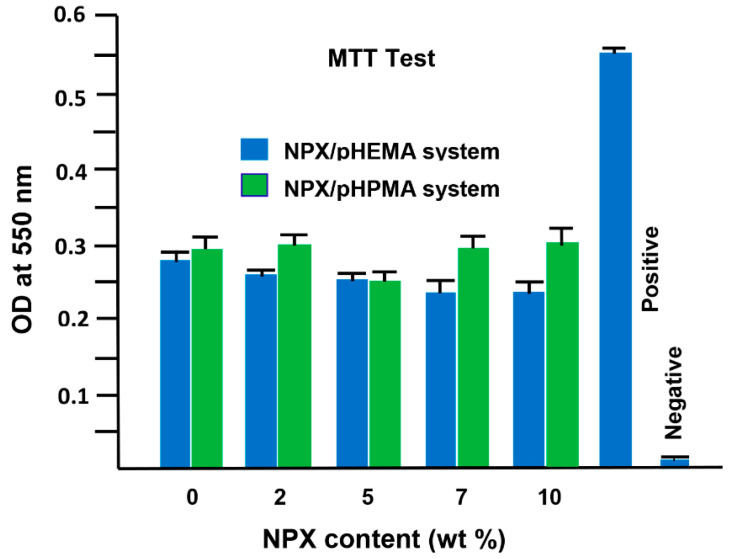
Effect of NPX content in NPX/pHEMA and NPX/pHPMA drug carrier systems on Ca9-22 cells adhesion

**Figure 10:**
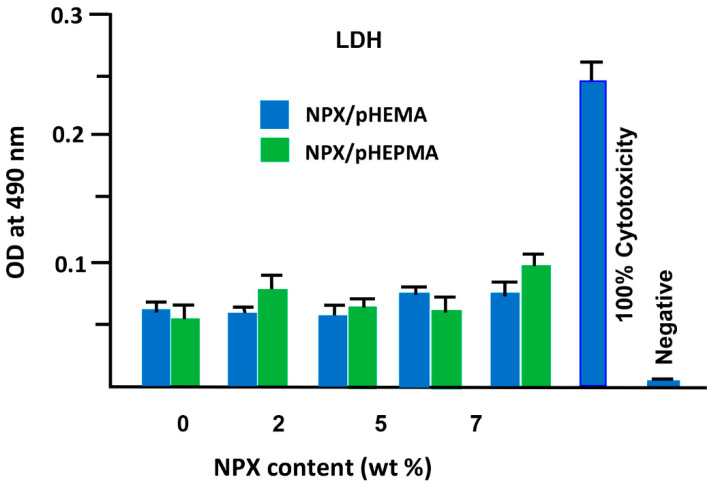
Effect of NPX content in NPX/pHEMA and NPX/pHPMA drug carrier systems on Ca9-22 cells cytotoxicity.

As shown in Figure 9 (in blue), the NPX/pHEMA drug-carrier system with different NPX contents presents, in general, a good adhesion compared to the negative (untreated tissue culture plate) and positive (tissue culture plate treated for cell adhesion) control used in this study. However, the NPX amount incorporated in the pHEMA in drug carrier system seemed to not significantly affect the cell adhesion when the Ca9-22 cells were treated with naproxen. These results were also confirmed by the LDH assay (Figure 10 in green). In addition, NPX/pHEMA drug carrier systems, as well as the pure pHEMA, induce low cytotoxicity compared to the negative and positive controls (2% Triton). Comparable results were also observed when the pHEMA was replaced by pHPMA in the drug carrier system, regardless of the range of the composition investigated (Figure 9 in blue and Figure 10 in green).

### 3.3. Swelling Behavior

**Figure 11:**
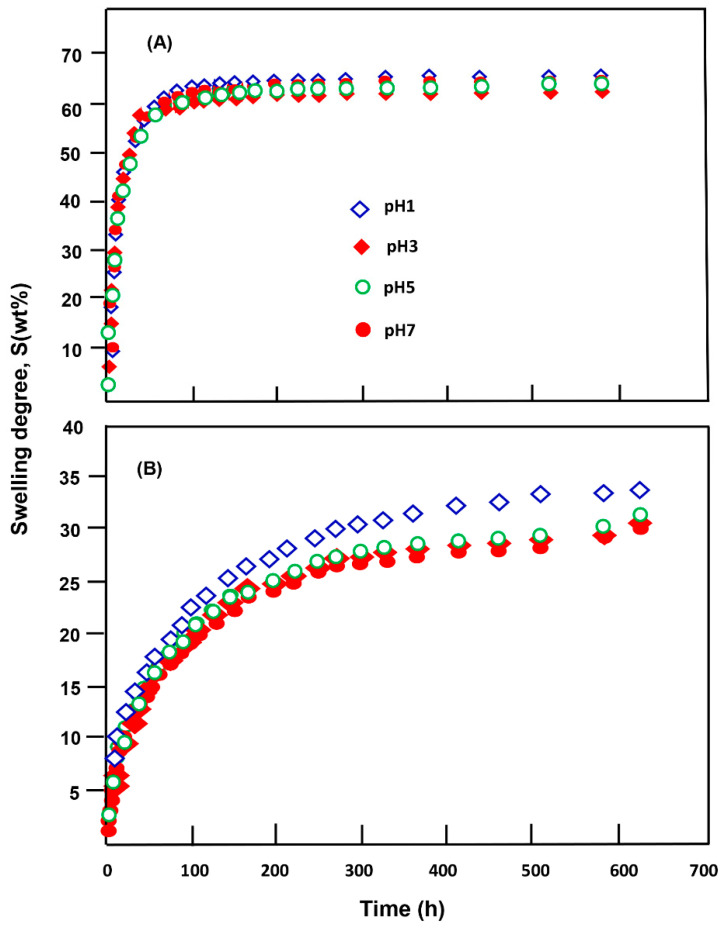
Variation of the swelling degree of (**A**) pHEMA, (**B**) pHPMA film samples versus time at 37 °C.

Figure 11 shows the variation of the swelling degree of pHEMA and pHPMA film samples versus time, and Table 2 collects the deducted swelling degree at equilibrium.

The comparison of the swelling degree values of these two polymer samples reveals that the absorption capacity of the pHEMA film is approximately double that of pHPMA, regardless of the pH of absorbed medium. This seems to be obvious and can be explained quite simply by the more hydrophilic character of the hydroxyethyl substitute belonging to the HEMA units, with regard to that of the hydroxypropyl of pHPMA, which contains an additional methyl. These results also reveal that, for both samples, the swelling capacity increased slightly when the pH medium decreased. This is probably due to the protonation of the oxygen of certain hydroxyl or carbonyl groups belonging to the monomeric units, thus increasing the hydrophilicity of the polymer. Generally, the swelling kinetics are used to investigate the diffusion of small molecules such as water through polymeric materials when immersed in a penetrating medium during a certain time. According to Comyn [46], the kinetics that govern the diffusion of small molecules through a polymer material are given by Equation (6)

(6) $$\frac{m_{t}}{m_{max}}=1-\sum_{n=0}^{\infty }\frac{8}{{\left(2n+1\right)}^{2}\pi^{2}}\exp\left[\frac{-D{\left(2n+1\right)}^{2}\pi^{2}t}{l^{2}}\right],$$

where *m_t_* and *m_max_* are the masses of the absorbed molecules during *t* time and at the maximum absorption (equilibrium), respectively. *D* and *l* are the diffusion coefficient with regard to the small molecules and the film thickness, respectively. For the short times of the initial stage of diffusion and when the *m_t_/m_max_* ratio is lower than 0.5, Equation (6) above takes the following expression:

(7) $$\frac{m_{t}}{m_{max}}=2\times {\left(\frac{D\times t}{\pi \times l^{2}}\right)}^{1/2},$$

in which, *D* can be deduced from the slope of the linear portion of the curve corresponding to the variation of *m_t_/m_max_* versus square root of time.

The fundamental equation of mass uptake by a polymer material is given by Equation (8) [47]:

(8) $$\frac{m_{t}}{m_{max}}=k\times t^{n},$$

where *n* exponent is the type of diffusion mechanism and *k* is the constant that depends on the diffusion coefficient and the film thickness. By analogy with Equation (7), *k* takes the following expression:

(9) $$k=\frac{2}{l}{\left(\frac{D}{\pi }\right)}^{n},$$

Equation (8) can be linearized by entering the logarithm of its two members as follows:

(10) $$\ln\left(\frac{m_{t}}{m_{max}}\right)=\ln k+n\ln t,$$

**Figure 12:**
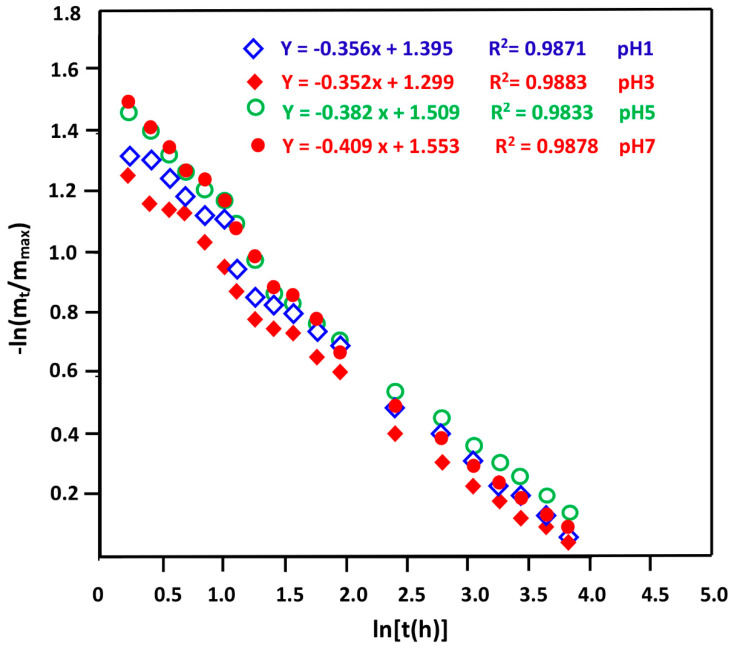
Variation of ln (*m_t_/m_max_*) versus ln (*t*) for the pHEMA material.

**Figure 13:**
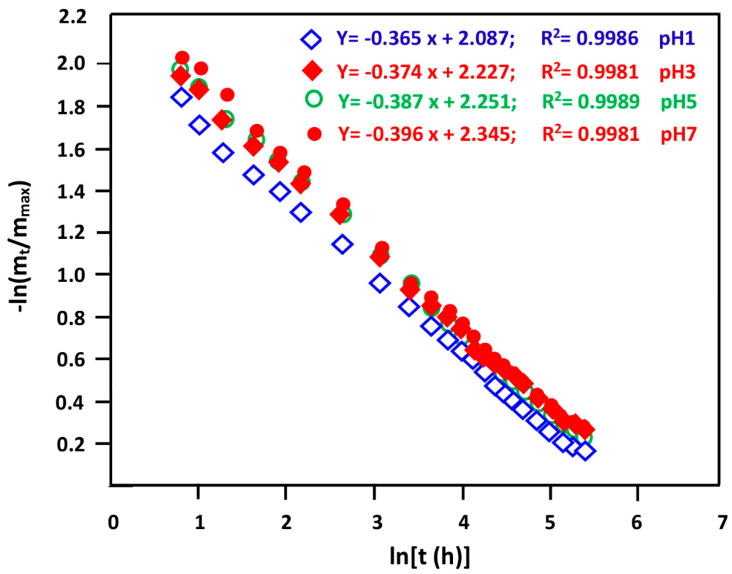
Variation of the ln (*m_t_/m_max_*) versus ln (*t*) for the pHPMA material.

The variation of ln(*m_t_*/*m_max_*) versus ln*t* for pHEMA and pHPMA materials is plotted in Figure 12 and Figure 13, respectively. Straight lines were obtained, indicating that the diffusion of water molecules through these polymer materials obeys the Fick’s model as long as their temperature in media (37 °C) is well above T_g_ (80 °C for pHEMA and 87 °C for pHPMA). This condition also indicates that the diffusion of water through the polymer film is purely and simply governed by a mechanical process and non-disturbed by a probable esterification reaction, which can occur in acidic media between the hydroxyl group contained in these polymers and the carboxylic group of Naproxen. The data of *n*, *D,* and *k* values deducted from these linear curves are gathered in Table 3. These results reveal, for both systems, an increase of the diffusion rate of water molecules when the pH of the medium increased. This property is highly valued in the field of drug delivery because this carrier is able to swell sufficiently and therefore delivers an appropriate amount of drug directly into the target organ (intestines, neutral pH medium).

As can be seen from these data, practically no change in the order of the water diffusion through each polymer material is observed, regardless the pH of medium, which is close to 0.40. The diffusion coefficient attributed to pHEMA material is higher than that of pHPMA, except those experimented in medium at neutral pH, which are both close to 0.80 mm^2^∙h^−1^. This is probably due to the decrease of the affinity between pHPMA and water caused by the hydropropyl group of the substitute (less hydrophilic) compared to that of hydroxyethyl group of pHEMA (more hydrophilic). These data also reveal that, for the pHEMA material, the *D* value decreased when the pH of the medium increased. In this same pH order, this parameter increased for the pHPMA.

### 3.4. Drug–Polymer Interactions

The drug–polymer Flory–Huggins interaction parameter denoted $\chi_{d,p}\;$gives an important idea on the chemical affinity and the magnitude of the adhesion force between the drug and the polymer carrier through its sign and its absolute value, respectively. According to the Flory–Huggins theory [48], a negative value of $\chi_{d,p}\;$indicates miscibility of a drug carrier system, and a positive value indicates its immiscibility. The $\chi_{d,p}$ values of the drug carrier systems involving NPX and pHEMA on the one hand and NPX and pHPMA on the other hand were estimated using the data of Table 4 and Equation (11) [49]:

(11) $$\frac{\Delta H_{f}}{R}\left(\frac{1}{T_{f}}-\frac{1}{T}\right)=\ln v_{d}+\left(1-\frac{1}{\lambda }\right)v_{p}+\chi_{d,p}v_{p}^{2},$$

where Δ*H_f_* and *T_f_* are the enthalpy of fusion of Naproxen and the melting temperature of the pure drug. *R* is the gas constant, and *T* is the measured solubility temperature for a volume fraction *v* with subscripts *d* and *p* denoting drug and polymer, respectively. λ is the ratio of the molar volumes of the drug and polymer. $\chi_{d,p}$ is the drug–polymer Flory–Huggins interaction parameter. The results obtained are gathered for comparison in Table 5.

As it can be seen from these results, the values of $\chi_{d,p}$ are all negative regardless of the drug carrier system and its composition. According to the Flory–Huggins theory, a negative value of $\chi_{d,p}\;$ indicates the miscibility of the drug carrier system. These data also reveal that the values of $\chi_{d,p}\;$ increase with the NPX loading incorporated into the polymer. This means an increase in the affinity of NPX molecules with regard to the polymer when the drug loaded in the drug carrier system increased. This seems to be obvious because the density of hydrogen bonds between the hydroxyl groups of pHEMA or pHPMA and the carbonyl of the carboxyl group of NPX increases with the drug load in the drug carrier system. This leads to an increase of the attraction forces between these two components.

The comparison of the values $\chi_{d,p}$ of the NPX/pHPMA system with those of the NPX/pHEMA system indicates a slight increase in the interactions between NPX and pHPMA compared to those between NPX and pHEMA, whatever the composition of the mixture studied. These results also reveal that the more the amount of NPX increases in the drug carrier system, the greater the absolute value of the difference between the Flory–Huggins interaction parameters of the NPX/pHEMA and NPX/pHPMA drug systems, $\Delta \left(\chi_{d,p}\right)$ also increases.

### 3.5. In Vitro Release Dynamic of NPX

#### 3.5.1. Release Kinetics of NPX

**Figure 14:**
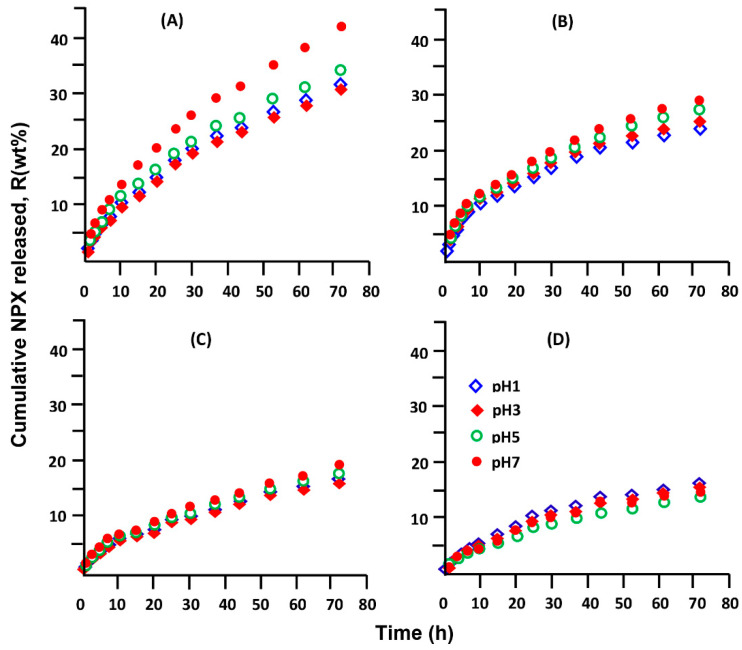
Release kinetics of NPX from (**A**) NPX/pHEMA2, (**B**) NPX/pHEMA5 (**C**) NPX/pHEMA7, (**D**) NPX/pHEMA10 drug carrier system at different pHs.

**Figure 15:**
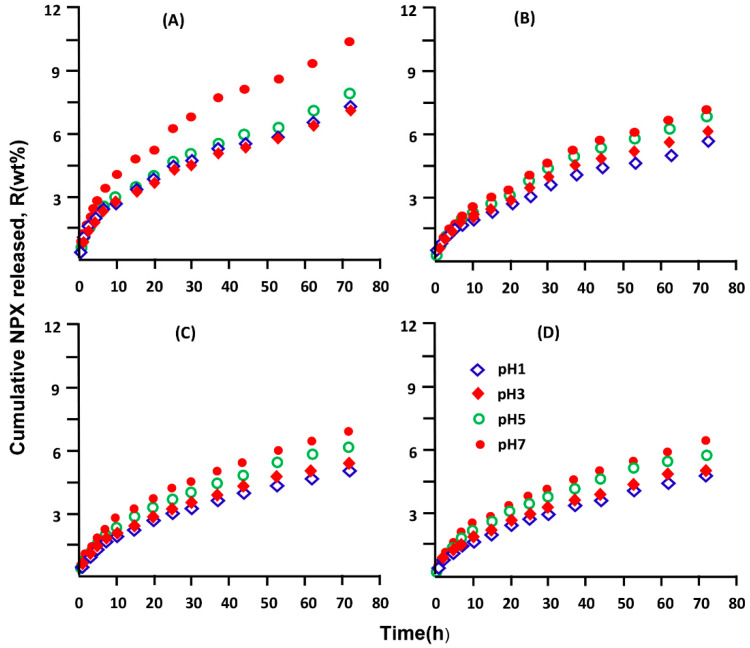
Release kinetics of NPX from (**A**) NPX/pHPMA2, (**B**) NPX/pHPMA5 (**C**) NPX/pHPMA7,(**D**) NPX/pHPMA10 drug carrier system at different pHs.

The release dynamic of NPX from NPX/pHEMA and NPX/pHPMA drug carrier systems with different compositions are shown in Figure 14 and Figure 15, respectively. As it can be seen from the curve profiles obtained for both systems, the maximum percentage of NPX released is reached with the drug carrier systems containing 2 wt% of NPX content. The comparison between the NPX release capacities for these two different systems during 72 h of the release process reveals that the NPX/pHEMA shows the best performance. Indeed, for the NPX/pHEMA2, a maximum of 42 wt% of NPX was released in neutral pH medium during this period and about 31 wt% in acidic media (pH 1 and 3), while for the NPX/pHPMA2, only 10.5% wt% of NPX was released in neutral pH medium and 7.4% in acidic media (pH 1 and 3) during the same period. This represents a reduction in NPX release dynamics of about a quarter. The decrease in NPX release dynamics observed by replacing pHEMA by pHPMA appears to be due to a dramatic decrease in the hydrophilicity of the carrier polymer caused by the additional methyl group in the substituent. The decrease in hydrophilicity when passing from the pHEMA to pHPMA carrier reduces the degree of swelling as revealed in Table 2. This limits the water amount absorbed by the polymer carrier necessary for the dissolution of a significant part of NPX incorporated in this material. It was also observed from these same curves, for both systems, the behavior of NPX release versus time is characterized by two pseudo stable zones of the release dynamic. The first zone, which is rapid and short, is observed during about the first 4–7 h of the release process, depending on the nature of the drug-carrier used and the pH of the medium. The second zone, which is long and slow, is observed during the 65 h of the release process. The first step is mainly attributed to the leaching of a fraction of NPX particles deposited on the surface or slightly embedded in the sample film. The second step characterizes the steady state in which the release of NPX in the media is governed mainly by a material transfer mechanism.

#### 3.5.2. Enhancement of NPX Solubility

The improvement of the solubility of NPX in water is an integral part among the objectives targeted by this investigation. To reach this goal, a comparison between the solubility of NPX in its powder form and that incorporated in the NPX/pHEMA and NPX/pHPMA drug carrier systems was carried out at 37 °C. An excess amount of NPX powder was dissolved under continuous stirring in a known volume of water maintained at 37 °C until the appearance of a stable precipitate, indicating the supersaturation of the solution. The solution was then filtered through a Whatman filter number 1. The solubility of NPX was determined by means of UV-visible spectroscopic analysis. Two solutions of pH 1 and 7 were prepared, and the results obtained are gathered in Table 6. The maximum NPX amount dissolved in these media was deducted from the maximum release of this medication from the NPX/pHEMA and NPX/pHPMA drug carrier systems, and the results obtained are also grouped for comparison in this table. The comparison of the maximum solubility data obtained reveals that the pHEMA is much more efficient than the pHPMA used as supports. These data also reveal an enhancement of the solubility of NPX in pH media 1 and 7, in which the NPX/pHEMA system was able to dissolve 2.60-fold that of that of because the NPX amount dissolved from the NPX/pHEMA10 system is more than 1.34 times in pH medium 1 and 2.60 times in neutral pH from the NPX/pHPMA system. The comparison of the NPX solubility data obtained by direct dissolution of the powder with that deduced from the release process involving these two polymers reveals a marked improvement when this drug is incorporated in the molecular state in one of these two polymers. These results also show that the pHEMA used as a support is more efficient than the pHPMA in increasing the solubility of NPX in water. For example, in neutral pH medium, pHEMA was able to improve the solubility of this medication by 3.32 times that of its direct dissolution as powder and 2.33 times in pH medium 1, while pHPMA increased this solubility by only 1.28 and 1.74 times in pH media 7 and 1, respectively.

#### 3.5.3. Surface Morphology

**Figure 16:**
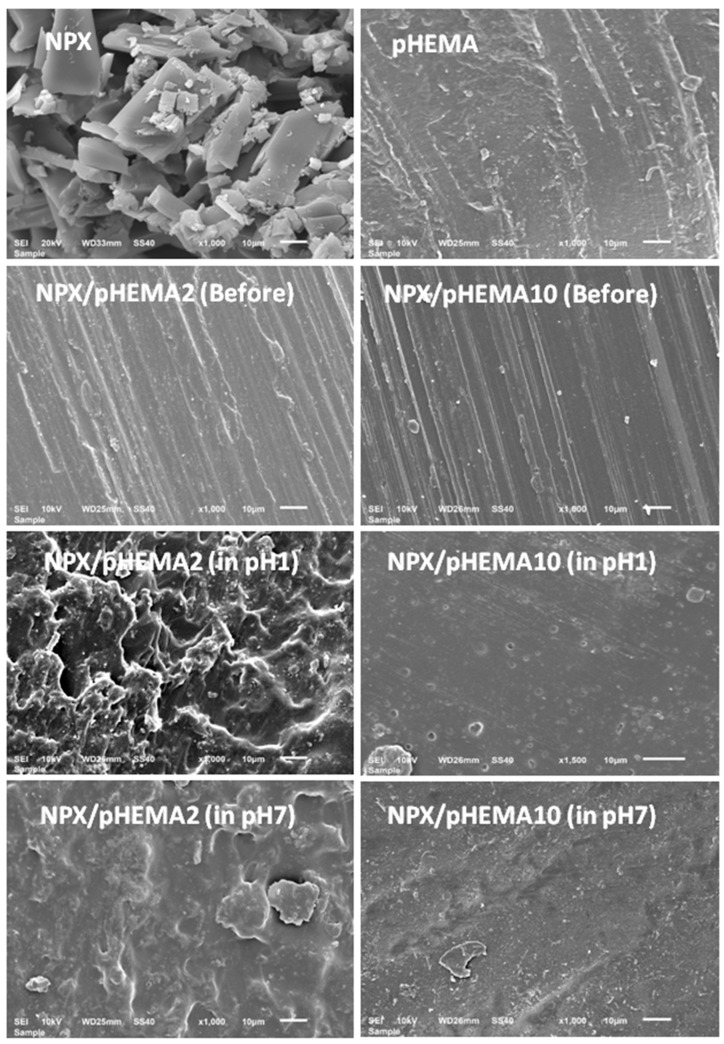
SEM images of NPX powder and surface morphology of virgin pHEMA, NPX/pHEMA2, and NPX/pHEMA10 film samples before and after the NPX release process in media pH 1 and 7.

**Figure 17:**
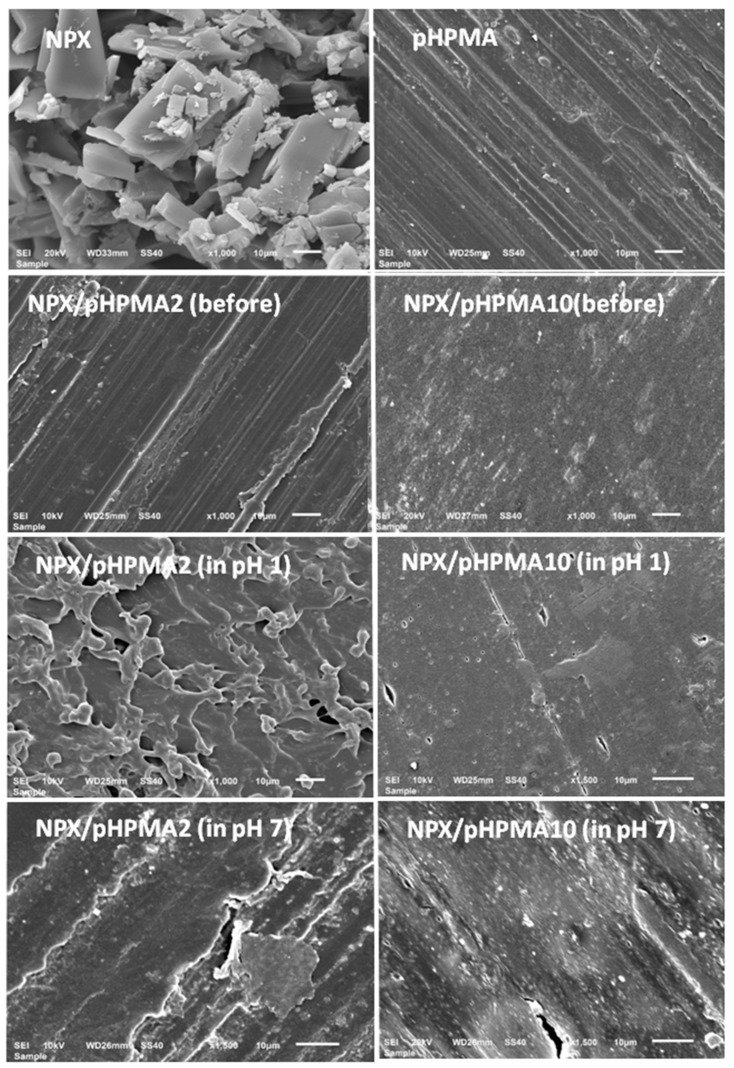
SEM images of NPX powder and surface morphology of virgin pHPMA, NPX/pHPMA2, and NPX/pHPMA10 films samples before and after the NPX release process in media pH 1 and 7.

Figure 16 groups the micrographs of NPX powder, virgin pHEMA, NPX/pHEMA2, and NPX/pHEMA10 film samples before and after the release process in media pH 1 and 7 chosen among the most significant images. The NPX image shows crystal particles aggregated into defined geometric shapes resembling piles of rubble from houses destroyed by an earthquake. These aggregates, which are sized between 3 µm × 3 µm × 2 µm and 50 µm × 25 µm × 6 µm, show smooth and homogeneous morphology surfaces, while the micrograph of the virgin pHEMA film presents roughness on the surface, which is probably due to the film preparation. NPX/pHEMA systems containing 2 wt% and 10 wt% NPX contents before the release process show comparable morphology surfaces, in which the observed obliquely aligned parallel grooves mark the surface of the mold where they were prepared. These same samples observed after the NPX release process in pH 1 and 7 media exhibit surface morphologies very marked by the very hollow relief and cavities, thus revealing the large amount of NPX released and also show a significant degree of swelling of the film notably in pH medium 1. Regarding the NPX/pHPMA drug carrier system, as shown in Figure 17, the images are practically comparable to those of the system involving pHEMA as carrier are observed. Indeed, the surfaces of the samples before the drug release process as for the blank carrier show the same type of grooves, except that with 10 wt% NPX, in which they are less marked. This reveals that the surfaces of the two carriers involved in the drug carrier system behave substantially the same during the drug release and show no particular mark distinguishing one or the other polymer.

#### 3.5.4. Diffusion Behavior of NPX

The diffusion behavior of NPX from NPX/pHEMA and NPX/pHPMA drug carrier systems was investigated. According to Lin et al. [52], for a percentage less than 60 wt% of a substance released from the initial amount incorporated into a material, the diffusion of this substance in its liquid state through this material follows a Fickian model, as long as, in this investigation, the limit of the percentage of NPX released is far from being reached whatever the drug carrier system and the composition. Fick model is therefore applicable to describe the diffusion behavior of NPX from the polymer matrix [53]. The equation resulting from the Fickian model is given by Equation (12) [54,55,56]

(12) $$\frac{m_{t}}{m_{o}}=k\sqrt{t},$$

where $m_{t}/m_{o}$ is the fraction of drug released, *t* is the release time, and *k* is a constant characteristic of each sample.

**Figure 18:**
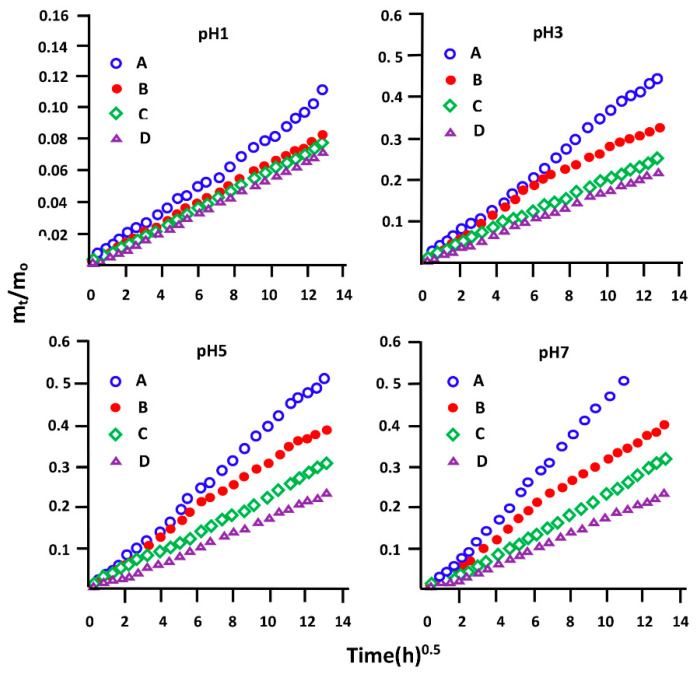
Diffusion behavior of NPX through (A) NPX/pHEMA2, (B) NPX/pHEMA5 (C) NPX/pHEMA7, (D) NPX/pHEMA10drug carrier system with different NPX contents in different pH media.

**Figure 19:**
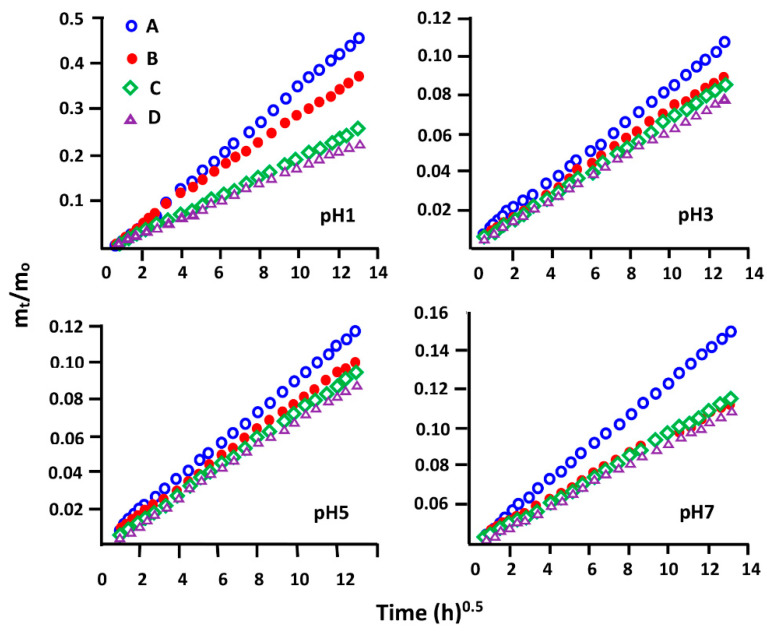
Diffusion behavior of NPX through (A) NPX/pHPMA2, (B) NPX/pHPMA5 (C) NPX/pHPMA7, (D) NPX/pHPMA10drug carrier system with different NPX contents in different pH media.

If the drug released from the drug carrier system obeyed the Fick diffusion model, the graph showing the change in the fraction of drug released $m_{t}/m_{o}$ versus the square root of time would give a straight line with a slope *k*. Under these conditions, the value of the diffusion coefficient (*D*) will then be deduced from Equation (13) [57]:

(13) $$k={}^{\prime }\sqrt{\frac{D}{\pi \times l^{2}}},$$

where *l* is thickness of film, from which the drug is released. The *k* and *D* values were calculated from the data of Figure 18 and Figure 19 using Equations. (11) and (12) and the results obtained are gathered in Table 7.

As it can be seen from these data, all the *R^2^* values are close to unity. This indicates that the data correspond well to the linear regression of these curve profiles. These results also indicate that the NPX release behavior from both NPX/pHEMA and NPX/pHPMA systems follows a Fickian model with an order of 0.5. The higher the value of k, the higher the diffusion coefficient and, therefore, the faster the rate of the drug diffusion through the carrier. In general, the *k* and *D* values increased with the pH medium regardless of the drug carrier system used. This can be explained by the solubility of NPX, which becomes more soluble in media of higher pH. Knowing that the pKa of Naproxen is equal to 4.19 [58], the solubility of this drug increases with increasing pH of the medium due to the passage of the carboxylic acid group towards the carboxylate salt group that is more soluble in water, notably when the pH medium becomes equal to or greater than the pKa. In addition, it can also be seen in Figure 18 and Figure 19, as the amount of NPX increases, the D value decreases. This can be explained by two main factors that can intervene simultaneously in the management of the drug release process: (i) the increase in the viscosity of the medium, which reduces the rate of diffusion and (ii) the presence of an NPX excess not soluble in the polymer matrix, which hinders the passage of soluble molecules during the diffusion process.

#### 3.5.5. Effect of the Initial NPX Amount

**Figure 20:**
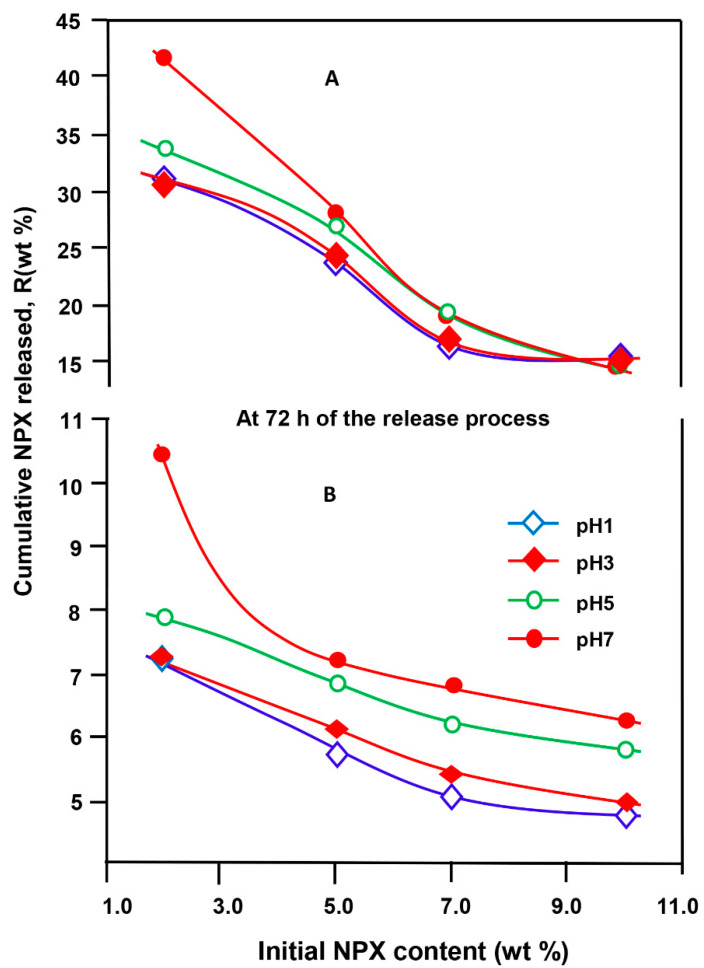
Cumulative NPX released from (A) NPX/pHEMA and (B) NPX/pHPMA drug carri-er systems at 72 h of the release process.

The influence of the initial NPX amount loaded in pHEMA and pHPMA carriers on the dynamic release of this medication from the corresponding drug-carrier systems was studied at a selected period of 72 h of the release process. The results obtained for NPX/pHEMA and NPX/pHPMA systems are plotted for comparison in Figure 20. As can be observed from these curve profiles, the two drug carrier systems have practically the same trends, in which the release dynamic decreased, passing through a reflection point at 6.0 wt% of NPX common for all samples and then stabilizes or tends to stabilize when the percentage of NPX is greater than 7.0 wt%.

A more rapid decrease in the release dynamic is also observed on these profiles in neutral pH medium when the initial NPX loaded in the drug carrier systems was less than 5 wt%. The decrease in the NPX released observed in all pH media, when the drug content in the polymer matrix increased, is mainly due to the limited solubility of this drug inside the drug carrier system and to the increase of the viscosity of the solution inside the polymer matrix. Indeed, dissolving loads greater than 5 wt% seems to be difficult, especially in acidic pH media.

#### 3.5.6. Effect of pH Medium

**Figure 21:**
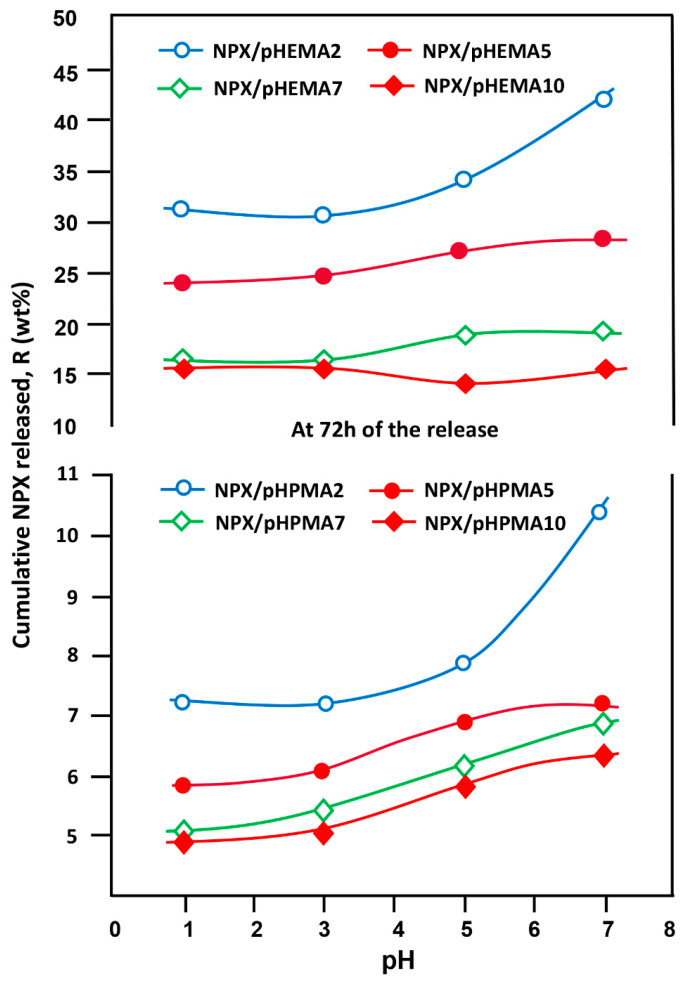
Variation of the NPX released from NPX/pHEMA and NPX/pHPMA drug carrier systems versus the pH of medium taken at 72 h of the release process.

The impact of the pH of the medium on the release dynamic of NPX from NPX/pHEMA and NPX/pHPMA systems was carried out at 72 h of the release process, and the results obtained are plotted in Figure 21. These curve profiles reveal comparable dynamics of the NPX released by the two drug-carrier systems regardless of the time period. Pseudo-stability of the release dynamics is observed for all samples in very acidic media (pH 1 and 3), then a slight increase or decrease depending on the initial NPX amount incorporated in the polymer matrix is observed at higher pH (5 and 7), except that containing the lowest NPX load (2 wt%), in which the release dynamic rapidly increased. The pseudo-reproducibility of the behavior of the drug release dynamics at different periods for these two systems shows that the transfer of NPX from the polymer material is mainly handled by a stable, purely mechanical process. The increase in the release dynamic with the pH of the medium is mainly due to the increase of the solubility of Naproxen in neutral pH media inside the polymer matrix. Indeed, as previously revealed from the results of Table 6, the solubility of NPX increased dramatically when the media pH increased. These results were also observed by Kumar et al. [59] and attribute the low solubility of Naproxen in lower pH media to its unionization. These same authors add that the unionization of the drug can facilitate its permeability through the polymer material, but drug solubility is the limiting factor.

#### 3.5.7. Performance of XPN/pHEMA and NPX/pHPMA Drug Carrier Systems

As it was noted in Section 3.5.1, for both the drug carrier systems, it was revealed that the release behavior of NPX versus time followed two main stages regardless of the composition and the pH of the medium. Each stage is characterized by a zone, in which the release dynamic of NPX passes by pseudo stability. The rate of NPX released during the corresponding period was taken from the slope of the pseudo linear curve, and the data obtained are illustrated for NPX/pHEMA and NPX/pHPMA systems in Table 8 and Table 9, respectively, noting that the cumulative percentage of the drug released during each period was calculated by multiplying the rate by time. Knowing that, for a system to be effective in the field of drug delivery, it must be able to uniformly deliver an appropriate amount of this drug in the intestines (neutral pH) and in the stomach (pH = 1–3). On this basis, the performance of these two systems on the NPX release was founded, and the results obtained are summarized for NPX/pHEMA in Table 8 and for NPX/pHPMA in Table 9. These data reveal that the two systems containing 2% by weight NPX appear to be the most effective of all the others because NPX/pHEMA2 drug-carriers were able to release 28.68 wt% of NPX uniformly during 65 h of the release process into a neutral medium with a release rate of 0.441 wt%·h^−1^. In contrast, only 21.32 wt% was released uniformly (0.328 wt%∙h^−1^) during this same period into the acidic medium (pH = 1). On the other hand, during the same period, the drug carrier system involving the pHPMA2 was able to release uniformly 6.83 wt% of NPX in neutral pH with a rate of 0.102 wt%∙h^−1^; at the same time, only 4.62 wt% was released in medium with pH = 1 with a constant rate of 0.069 wt∙h^−1^. In general, the comparison of the performances of these two systems reveals that involving the pHEMA appears to be the most efficient.

As it can be seen from the results of Table 9, the NPX/pHPMA2 drug carrier system appeared to be the best performing system in terms of the percentage of NPX released into the medium at neutral pH over the longest period. Indeed, this system was capable to release uniformly the greatest percentage of NPX (28.67% by weight) in the medium at neutral pH (intestines) with a release rate of 0.44 wt%∙h^−1^ for 67 h of the release process. During this time, only 21.32 wt% of this drug was released into the medium at pH = 1 (similar to that of in the stomach), with a constant rate of 0.33 wt%·h^−1^. Concerning the system involving the pHPMA as a carrier, as in the case of that of the NPX/pHEMA system, the most efficient is that initially containing 2 wt% of NPX (NPX/pHPMA2). Indeed, 6.83 wt% NPX was released uniformly (0.102 wt%·h^−1^) from this system in the neutral pH medium and 4.62 wt% slowly (0.069 wt%·h^−1^) in acidic medium (pH1) during the same period. Thus, regardless of the polymer used as a carrier in this work, the most efficient system is the one that contains the least NPX load. Finally, the addition of a methylene group on the substituent of the hydroxyl ethyl methacryloyl unit of pHEMA had the effect of reducing by more than four times the percentage of NPX released, as well as its release rate in the various media invested. This can be attributed to the reduction in the hydrophilicity of the polymer upon switching from pHEMA to pHPMA.

#### 3.5.8. Distribution of NPX Released on Target Organs

According to Belzer et al. [60], the mean total gastrointestinal transit time (GITT) is between 53 and 88 h divided into three main stages: (i) gastric transit (pH 1.5–3, 5), which lasts between one and 4 h; (ii) intestinal transit (pH 7–9), which varies between 4 and 12 h; (iii) transit in the colon (pH 5–7), which lasts between 48 and 72 h. Taking into account the pH of the medium and the GITT, it was possible to estimate approximately from the data in Table 8 and Table 9 the distribution of the percentages of cumulative NPX released in different organs and the mean stomach/digestive organ ratio (SDOR) (Equation (14)), independently of the effects of enzymes and microorganisms.

(14) $$SDOR(wt\%)=\frac{r_{s}}{r_{si}+r_{c}}\times 100$$

where $r_{s}$, $r_{si}$, and $r_{c}$ are the percentages of NPX released in the stomach, small intestine, and colon, respectively, during a certain transit time.

The results obtained are gathered for comparison in Table 10. These data reveal that both the drug carrier systems containing 2 wt% of NPX are the most efficient because the NPX/pHEMA2 drug carrier systems are able to reduce the NPX amount released in the stomach to 3.18 wt% of the total amount released for the fast GITTs and 14.85 wt% for the slow GITTs, and 4.41 wt% and 14.83 wt% for the NPX/pHPMA2 system.

## 4. Conclusions

To conclude this work, we can say that the objectives of this work have been achieved. Indeed, the comparison between the physicochemical properties of pHPMA with those of pHEMA revealed properties slightly inferior to those of pHEMA necessary for the admission of pHPMA as a carrier in the drug delivery domain. The miscibility of NPX with pHEMA and pHPMA binary systems, in which the NPX is distributed uniformly in its molecule state, are proven in all compositions by the FTIR method through the presence of hydrogen bonds between their components. This miscibility was also confirmed by the DSC method through the shift toward the low temperatures of the *Tg* of the polymer, the disappearance of the melting temperature of NPX in the mixture, and by XRD through the disappearance of the signals characterizing the crystalline structure of NPX.

The cell adhesion essay and cytotoxicity test of pure polymers and drug carrier systems revealed that the NPX/PHPMA system, as well as the NPX/pHEMA system with compositions, generally exhibit good adhesion compared to the negative and positive controls used in this study. In addition, these two systems, as well as their pure polymers, induce low cytotoxicity compared to the negative and positive controls.

The swelling study of pHEMA and pHPMA carriers revealed that the presence of additional methylene group in the substituent of the HPMA unit of pHPMA caused the swelling capacity to drop to half that of pHEMA. The determination of the Flory–Huggins interaction parameters of the NPX/pHEMA and NPX/pHPMA binary systems reveals greater interactions between the components of NPX/pHEMA system at compositions equal to or less than 5 wt% NPX; on the other hand, they are greater for NPX/pHPMA at compositions greater than 5 wt% NPX.

The “in vitro” study of the release dynamic of Naproxen from NPX/pHEMA and NPX/pHPMA drug carrier systems revealed that the higher percentage of NPX released was obtained from each polymer carrier in neutral pH medium, and the diffusion of water and NPX solution trough these polymer matrices also obeys the Fickian model with a kinetics order close to 0.5, regardless of the pH of the medium. It was also found that the less the mass percent of NPX in the composites, the better its release will be. The comparison between the two drug carrier systems revealed that the pHEMA leads to the best performance in the release dynamic of NPX.

Regarding the Naproxen solubility in water, the results deducted from the “in vitro” study of NPX/pHEMA10 and NPX/pHPMA10 drug carrier systems reveal a very significant improvement in the solubility of NPX in media pH1 (2.33 times, 1.43 times) and 7 (3.32 times, 2.60 times), respectively, compared to those obtained by direct dissolution of Naproxen powder.

According to Belzer, the approximate estimation of the distribution of the percentages of cumulative NPX released in different organs and the mean stomach/digestive organ ratio, independently of the effects of enzymes and microorganisms, revealed that both drug carrier systems containing 2 wt% of NPX are the most efficient because the NPX/pHEMA2 drug carrier systems are able to reduce the NPX amount released in the stomach to 3.18 wt% of the total amount released for the fast GITTs, 14.85 wt% for the slow GITTs, and 4.41 wt% and 14.83 wt% for the NPX/pHPMA2 system. Although pHEMA seems to be the more performing carrier of the two polymers when administered orally (requiring a relatively large amount of drug absorbed at neutral pH), pHPMA combined with a small amount of medication (2 wt%) can also be used if the purpose is the application on the skin surface or as contact lenses to treat certain diseases of the surface of eyes caused by viruses, bacteria, parasites, and fungi because the eyes absorb only a tiny amount of the drug dissolved in a neutral medium. In this case, a regular release of small amounts of drug for as long as possible is desirable in order to limit the frequency of administration of the drug by this route, providing more comfort to the patient.

## Figures and Tables

**Table 1:** Preparation conditions of NPX/pHEMA and NPX/pHPMA composites.

Drug-Carrier System	HEMA (g)	HPMA (g)	NPX (g)	NPX (wt%)	Camphorquinone (g)
NPX/pHEMA2	5.00	-	0.102	2.0	1.0
NPX/pHEMA5	5.00	-	0.263	5.0	1.0
NPX/pHEMA7	5.00	-	0.376	7.0	1.0
NPX/pHEMA10	5.00	-	0.555	10.0	1.0
NPX/pHPMA2	-	5.00	0.102	2.0	1.0
NPX/pHPMA5	-	5.00	0.263	5.0	1.0
NPX/pHPMA7	-	5.00	0.376	7.0	1.0
NPX/pHPMA10	-	5.00	0.555	10.0	1.0

**Table 2:** Swelling capacity of pHEMA and pHPMA film samples in different pH of medium.

Film Sample	Swelling Degree at Equilibrium			
	pH			
	1	3	5	7
pHEMA	66.21	64.14	62.50	62.45
pHPMA	34.22	33.36	32.88	32.06

**Table 3:** Diffusion parameters of water at different pHs through pHEMA and pHPMA materials.

Polymer Sample	*n*				*k*				*D* (mm^2^∙h^−1^)			
pH	*1*	*3*	*5*	*7*	*1*	*3*	*5*	*7*	*1*	*3*	*5*	*7*
pHEMA	0.36	0.35	0.38	0.41	4.03	3.67	4.52	4.73	1.16	1.24	1.06	0.80
pHPMA	0.37	0.37	0.39	0.40	8.06	9.27	9.50	10.43	0.54	0.44	0.51	0.79

**Table 4:** Some characteristic data of pHEMA, pHEPMA, and Naproxen taken at 25 °C.

Compound	Density (g∙mL^−1^)	Hansen Solubility Parameter (KPa^1/2^)	Molar Volume (mL∙mol^−1^)	Δ*H_f_* (kJ∙mol^−1^)	*T_f_* (°C)
pHEMA	1.25		103.8	-	-
pHPMA	1.33		108.2	-	-
Naproxen	1.27 ^c^	21.62 ^c^	157.3^c^	31.50 ^a,b^	154.6 ^a,b^

^a^ Ref. [50]; ^b^ Ref. [34]; ^c^ Ref. [51].

**Table 5:** Comparative values of the Flory–Huggins parameters of the NPX/pHEMA and NPX/pHPMA drug carrier systems determined at 25 °C using Equation (9).

Drug-Carrier System	*χ_d,p_*	Drug-Carrier System	*χ_d,p_*	Δ(*χ_d,p_*)
NPX/pHEMA2	−0.27	NPX/pHPMA2	−0.30	0.03
NPX/pHEMA5	−1.28	NPX/pHPMA5	−1.33	0.05
NPX/pHEMA7	−1.72	NPX/pHPMA7	−1.77	0.05
NPX/pHEMA10	−2.26	NPX/pHPMA10	−2.32	0.06

**Table 6:** Maximum solubility of NPX dissolved in pH media 1 and 7 at 37 °C.

System	pH 1	pH 7
NPX powder	29.51	61.68
NPX/pHEMA10	68.84	205.04
NPX/pHPMA10	51.28	78.77

**Table 7:** Summary of diffusion data NPX through NPX/pHEMA and NPX/pHPMA drug carrier systems.

pH	System	*k*∙10^2^ (h^−1/2^)	*R* ^2^	*D*∙10^3^ (mm^2^·h^−^^1^)	System	*k* (h^−1/2^)	*R* ^2^	*D* 10^3^ (mm^2^·h^−1^)
1	NPX/pHEMA2	3.56	0.9988	6.161	NPX/pHPMA2	0.85	0.9991	0.386
3	NPX/pHEMA2	3.54	0.9976	6.642	NPX/pHPMA2	0.82	0.9994	0.359
5	NPX/pHEMA2	3.96	0.9983	8.830	NPX/pHPMA2	0.91	0.9996	0.401
7	NPX/pHEMA2	4.76	0.9990	13.239	NPX/pHPMA2	1.22	0.9995	0.636
1	NPX/pHEMA5	2.71	0.9950	3.628	NPX/pHPMA5	00.66	0.9996	0.270
3	NPX/pHEMA5	2.79	0.9950	3.678	NPX/pHPMA5	0.70	0.9994	0.262
5	NPX/pHEMA5	3.09	0.9977	4.240	NPX/pHPMA5	0.79	0.9997	0.278
7	NPX/pHEMA5	3.25	0.9978	5.343	NPX/pHPMA5	0.81	0.9994	0.396
1	NPX/pHEMA7	1.93	0.9998	1.724	NPX/pHPMA7	0.61	0.9997	0.172
3	NPX/pHEMA7	1.96	0.9994	2.446	NPX/pHPMA7	0.65	0.9994	0.198
5	NPX/pHEMA7	2.26	0.9985	3.017	NPX/pHPMA7	0.73	0.9998	0.262
7	NPX/pHEMA7	2.36	0.9941	3.597	NPX/pHPMA7	0.81	0.9999	0.348
1	NPX/pHEMA10	1.80	0.9980	1.338	NPX/pHPMA10	0.0032	0.9996	0.147
3	NPX/pHEMA10	1.81	0.9987	1.455	NPX/pHPMA10	0.0032	0.9996	0.147
5	NPX/pHEMA10	1.75	0.9976	1.254	NPX/pHPMA10	0.0036	0.9996	0.167
7	NPX/pHEMA10	1.80	0.9977	1.6778	NPX/pHPMA10	0.0046	0.9997	0.205

**Table 8:** Percentage of NPX released and instantaneous release rate of NPX from NPX/pHEMA system with different compositions.

System	pH	SZ (h)	RNR (wt%∙h^−1^)	CNR (wt%)	LR (R^2^)	System	SZ (h)	RNR (wt%∙h^−1^)	CNR (wt%)	LR (R^2^)
NPX/pHEMA2	1	0–7	1.001	07.01	0.978	NPX/pHEMA7	0–4	0.776	3.104	0.985
		7–72	0.328	21.32	0.976		4–72	0.171	11.29	0.979
	3	0–7	0.964	0.138	0.972		0–4	0.803	3.21	0.982
		7–72	0.336	21.84	0.975		4–72	0.172	11.35	0.973
	5	0–7	1.124	7.87	0.967		0–4	0.783	3.13	0.985
		7–72	0.366	23.79	0.977		4–72	0.209	13.79	0.981
	7	0–7	1.290	9.03	0.980		0–4	0.636	2.54	0.988
		7–72	0.441	28.67	0.982		4–72	0.223	14.72	0.987
NPX/pHEMA5	1	0–7	0.969	6.783	0.977	NPX/pHEMA10	0–4	0.687	2.75	0.951
		7–72	0.229	14.89	0.938		4–72	0.185	12.58	0.921
	3	0–7	0.993	6.95	0.986		0–4	0.668	2.67	0.973
		7–72	0.233	15.15	0.943		4–72	0.180	6.80	0.933
	5	0–7	1.081	7.57	0.975		0–4	0.718	2.87	0.977
		7–72	0.275	17.88	0.950		4–72	0.161	10.95	0.967
	7	0–7	1.013	7.09	0.971		0–4	0.605	2.42	0.990
		7–72	0.300	19.50	0.939		4–72	0.174	11.83	0.961

SZ: stability zone; RNR: rate of the NPX release; CNR: cumulative NPX released; LR: linear regression.

**Table 9:** Percentage of NPX released and instantaneous release rate of NPX from NPX/pHPMA system with different compositions.

System	pH	SZ (h)	RNR (wt%∙h^−1^)	CNR (wt%)	LR (R^2^)	System	SZ (h)	RDR (wt%∙h^−1^)	CNR (wt%)	LR (R^2^)
NPX/pHPMA2	1	0–5	0.330	1.65	0.966	NPX/pHPMA7	0–5	0.285	1.43	0.955
		5–72	0.069	4.62	0.977		5–72	0.076	5.09	0.975
	3	0–5	0.311	1.56	0.971		0–5	0.193	0.97	0.953
		5–72	0.069	4.62	0.980		5–72	0.052	3.48	0.973
	5	0–5	0.330	1.65	0.985		0–5	0.209	1.05	0.963
		5–72	0.077	5.162	0.982		5–72	0.057	3.82	0.976
	7	0–5	0.511	2.56	0.990		0–5	0.244	1.22	0.981
		5–72	0.102	6.83	0.972		5–72	0.064	4.29	0.977
NPX/pHPMA5	1	0–5	0.244	1.22	0.968	NPX/pHPMA10	0–5	0.184	0.92	0.929
		5–72	0.061	4.09	0.977		5–72	0.050	3.35	0.974
	3	0–5	0.250	1.25	0.960		0–5	0.193	0.97	0.952
		5–72	0.066	4.42	0.973		5–72	0.052	3.48	0.977
	5	0–5	0.279	1.40	0.963		0–5	0.224	1.12	0.944
		5–72	0.072	4.82	0.971		5–72	0.060	4.02	0.977
	7	0–5	0.285	1.43	0.955		0–5	0.240	1.20	0.954
		5–72	0.076	5.09	0.975		5–72	0.066	4.42	0.978

SZ: stability zone; RNR: rate of the NPX release; CNR: cumulative NPX released; LR: linear regression.

**Table 10:** Estimated distribution of the cumulative NPX released from NPX/pHEMA and NPX/pHPMA drug carrier systems on the principal digestive organs timed, according to Belzer approach.

Drug Carrier	Stomach		Small Intestine		Colon		SDOR	
System	Transit (wt%)		Transit (wt%)		Transit (wt%)		(wt %)	
Transit Time	Min	Max	Min	Max	Min	Max	Min (48)	Max (72)
	(1 h)	(4 h)	(4 h)	(12 h)	(48 h)	(72 h)		
NPX/pHEMA2	0.98	3.93	5.16	7.94	24.7	17.2	3.18	14.85
NPX/pHEMA5	0.98	3.92	4.05	5.44	14.4	17.1	5.04	14.46
NPX/pHEMA7	0.79	3.16	2.54	2.68	10.7	12.04	5.63	17.67
NPX/pHEMA10	0.68	5.42	2.42	2.09	8.35	11.83	5.94	28.02
NPX/pHPMA2	0.32	1.28	2.04	1.64	4.9	5.71	4.41	14.83
NPX/pHPMA5	0.25	0.99	1.14	1.13	3.65	5.09	4.96	13.73
NPX/pHPMA7	0.24	0.96	0.96	0.96	3.07	4.29	5.62	15.46
NPX/pHPMA10	0.22	0.87	0.96	0.97	3.17	4.42	5.06	13.9

## Data Availability

The data presented in this study are available on request from the corresponding author.
